# Gender Differences in Strategic Behavior in a Triadic Persecution Motor Game Identified Through an Observational Methodology

**DOI:** 10.3389/fpsyg.2020.00109

**Published:** 2020-02-04

**Authors:** Miguel Pic, Vicente Navarro-Adelantado, Gudberg K. Jonsson

**Affiliations:** ^1^Department of Specific Didactics, University of La Laguna, San Cristóbal de La Laguna, Spain; ^2^Human Behavior Laboratory, University of Iceland, Reykjavik, Iceland

**Keywords:** gender, triad, complexity, game, THEME

## Abstract

The main objective of the work is to address the effective behavior of girls and boys through Triadic Motor Games (TMG). A chasing game “The Maze” was applied on two class groups with a total of 42 players, 18 girls, and 24 boys, who were 12- and 13-year-old secondary school students. An observational methodology was adopted, with a nomothetic, punctual, and multidimensional design. We used a mixed registry system that two expert observers later applied through an observational methodology, obtaining sufficient record-quality levels. THEME was applied to detect temporary regularities, while cross-tabulations and growth trees were applied with the SPSS v.24 tool to reveal whether girls and boys played in similar or distinct ways. The fact that the specific decision groups within the physical education class are different for girls and boys (*p* < 0.005) is worth reflecting on. The game’s TMG complexity was addressed through roles and subroles, giving rise to a certain motor asymmetry in relation to gender, which is an expression of behaviors lacking in playful neutrality. Through a mixed-methods approach, a study was built using observational methodology that reveals more varied motor solutions in girls, while male behavior showed greater specialization of roles and subroles, and the linkage of these solutions with the favorable modification of the marker. Identifying relevant variables when playing TMG allows a better understanding of girls and boys by analyzing their relationships, which are sometimes paradoxical, in a practical context.

## Introduction

The game is a laboratory for the analysis of gender behavior, because playing is an activity that brings out patterns of action from a background of playful culture (Pellegrini et al., 2007); within this adaptive response, gender is constructed in a playful social relational context. Speaking about gender is evidence of a line of stereotypes (Knight, 2002) and asymmetries based on male supremacy (Travers, 2008; Chalabaev et al., 2013) expressed in contexts of wide social and sports impact, such as, for instance, in the Olympic Games (Pic, 2018a). It is through the application of motor games that the interactive expression that gives rise to each game is unleashed, among a set of motor communication models (Parlebas, 1981, 1988).

It would be difficult to conceive of the study of relationships while playing without contemplating the exercise of power (Caplow, 1956, 1959; Foucault, 1979; Connell, 2005) in communication models based on confrontation, where one team wins but another loses. There are some motor games that form the ludic heritage, such as the original balle assise game or its possible variants (Oboeuf et al., 2008; Lavega et al., 2011; Pic and Lavega-Burgués, 2019; Pic et al., 2019). Specifically, triadic motor games (TMG) have great situational complexity, but the question arises of what girls decide and what boys decide when playing in the same triadic situations. Knowing the motor behavior of male and female players in triads, based on observable responses, will reveal similarities or differences according to the gender of the players. In this way, it would be revealed whether this type of game deserves to have a different pedagogical treatment for girls and boys in a physical education class.

The increase in complexity and decisional dynamism (Araújo et al., 2014) of the triad can clarify our understanding of the triadic properties, which are non-existent in motor duels. The difference in the triad is that a coalition of the weak against the strong side is necessary, which is evidenced through the paradox; therefore, it is a paradoxical game. The question of whether there are gender differences in the game has been answered as a contribution of game and play to the socializing process (Lever, 1976), detecting that gender differences were due to the social context in which the game was developed (differentiation of roles, the interdependence between players, the size of the game group, the explicit explanation of objectives, the number of rules, and the formation of teams), the game of the boys being more complex (Balagué et al., 2013) than that of the girls (Lever, 1978). But perhaps this requires a specific interpretation of the structure of the game and the system of rules, because each game offers a specific context of situations (Gómez et al., 2014) and the rules channel the behavior in the game (Parlebas, 1981, 1988).

Closing the question, Evaldsson (2003) studies gender differences in sports games, and justifies them more by situationality; of course, it is in the situation where the variables that interest us converge. Certainly, Parlebas posits a motor communication where the “motor situation” is described as “a set of objective and subjective elements that characterize the motor action of one or more players in a given physical environment when performing a motor task” (1981, p. 220). When dealing with motor games, the motor situation brings together the task and its achievements, together with the emergence of an internal logic of the motor behaviors put into practice in an order of a strategic nature. As a consequence, the decisions of the players can be configured in T-patterns (Jonsson et al., 2010).

The game of persecution serves as a model facilitator of a structure capable of developing an internal logic derived from the conditions under which it is played and that comes from the rules. It is not a game of persecution where a player pursues others, which would have little strategic value, but a triad pursuit game in which there is simultaneous participation, which elevates the relationship to a high level of decisions and a greater complexity of those relationships. Consequently, the fact of the social relationship and the way of acting before it intensifies in the practice of the game. It is this internal structure of the game of persecution that nourishes and orders the mixed system of registration for observation, since in regulated games, it is a closed system due to the effect of the rules. This circumstance brings as a consequence a more solid method when looking for patterns with which to understand the behavior and gender differences when playing; in addition, it is recognized that the gender issue is linked to an empirical pattern and the order of power or forces that develop in the game (Bjerrum and Thorne, 2014). In this sense of motor communication and in its methodological transfer, the role in the game is taken as a decisional criterion of the system of rules, and it is the evidence consistent with the logic of their situations (Pic et al., 2018).

It should be noted that the style of game behavior is accessible to observation through the analysis of strategic behavior, thanks to the fact that it is about motor behavior. Thus, the strategic behavior becomes the differentiating element that deals with the motor problem that gives meaning to the achievements of the game actions. That is to say, the game has to be studied with rules based on the confrontation that justifies a winner. The behavior of boys and girls in confrontation games points to stereotyped forms of participation, where boys were more active and girls were more passive (Gutiérrez and García-López, 2012). However, it could be a mistake to make extensive generalizations of these results. This type of gender differences, unless proven by research, could be contrived and produced by cultures; being elevated to the category of cultural hegemony (Connell and Messerschmidt, 2005). It is possible to classify boys or girls because of their closeness to games and sports, with the known problem of learning and motivation (Ntoumanis et al., 2009). Opting for persecution games means reducing that level of learning and assuming a similar initial motivation, even if it is inevitable to separate the world from playful preferences. However, it should be noted that gender differences in this type of games were already noted by Sluckin (1981) and Navarro (1995).

In Pic et al. (2018) the decisional differences between girls and boys in different versions of the paradoxical game balle assise are verified (Guillemard et al., 1988; Parlebas, 1988; Oboeuf et al., 2008), describing game sub-roles (behaviors developed from a role) and the decision tree; the largest gender differences came from decisional efficiency in positive or cooperative (Dyson et al., 2004) and negative (Heider, 1946) valence behaviors in favor of boys (effect sizes, *d* = 0.46), in line with recent results (Pic and Lavega-Burgués, 2019). This previous research by Oboeuf et al. (2008) confirms the specificity and scope of the communication network (Parlebas, 1981) when confronting a duel motor game and in the game balle assise.

Sluckin (1981) found that boys’ commitment was four times higher than girls’ in a set of games, including persecution. Also, in boys and girls, at 8- and 10-year-old levels, and in similar recreational conditions, Navarro (1995, 2009) warns that one of the most relevant gender differences in 10-year-old girls with respect to boys was the development of secondary roles while playing against the roles that lead to success in a game of persecution; on the other hand, in the first level of 8-year-olds, that difference was not manifested. Specifically, reserving persecution roles for boys versus girls was a way to prioritize participation in motor games. Following the same methodological and theoretical trail, Pic et al. (2018) addressed the complexity of two motor games of different triads of the triadic census (Pic and Navarro, 2017; Pic and Navarro-Adelantado, 2019) through T-pattern analysis. A T-pattern is a powerful alternative to the discovery of hidden patterns based on events and temporal parameters in order to find a self-similar tree structure (Jonsson et al., 2006; Casarrubea et al., 2013, 2014; Pic, 2018b).

The use of mixed methods allows the study of the scenario of playful specificity due to the relevance of the temporal order of motor events. In recent history, the TMG (Parlebas, 2011; Navarro and Pic, 2016) temporal structure has been revealed in two forms of play with different properties (Pic et al., 2018), with greater decisional complexity found in the game “The Maze” than in “Three Fields.” When we study the responses of our students to an increase in complexity due to the triad, the emergence of new relationships would be favored, as well as encouraging strategic reflection in a spontaneous context. Revealing the existence of differences according to gender could make the teacher modify their methodologies or tasks in physical education classes. For this, the use of mixed methods was justified by observational methodology, based on the categories of roles and subroles (Parlebas, 1981, 1988) and the temporal structure of patterns.

The advantages of the use of mixed methods (Todd and Nerlich, 2004; Anguera et al., 2018), the integral vision of the object of study, the flexibility of the conceptual framework (Brannen, 1992; Johnson et al., 2007), and the inclusion of new dimensions, justify their selection for the study of motor triads, because they allow a problem to be addressed in its context and complexity. Specifically, the merger of purely quantitative aspects (e.g., the coding or control of data quality) with the use of qualitative aspects such as observational design and the use of an “*ad hoc*” registration system give the study a holistic view.

Based on the above considerations, the proposed study had two objectives:

1. Identifying the motor responses (descriptive and associative) of girls and boys through the development of TMG.
2. Determining the existence of gender differences when playing TMF through predictive models and T-patterns.

## Materials and Methods

### Study Design

For this study, a pre-experimental design was used (Bisquerra, 2004) composed of a single test applied to two non-equivalent class groups. More concretely, a quadrant III observational methodology was selected (Blanco-Villaseñor et al., 2003; Anguera and Hernández-Mendo, 2016). Specifically, a design was applied that was: (a) nomothetic, as data on different players were recorded; (b) punctual, because the observation were raised in a precise moment, and (c) multidimensional, since different dimensions (criteria) were taken into account (Anguera et al., 2011). The selection of a suitable design is essential in research, since the type of data (Bakeman, 1978) will determine the analysis technique, among other relevant decisions to achieve the research objectives.

### Participants

The number of players was 42, 18 girls (42.8%), and 24 boys (57.1%), between 12 and 13 years old (M = 12.5; DT = 1) from two secondary middle-class schools in the Canary Islands (Spain). The students played the same well-known motor game “The Maze,” distributed into two ecological (natural) class groups; in each center, there were two groups (group “1,” group “2”). Each group was divided into three teams composed of seven participants. Team A and B of group “1” consisted of four boys and three girls, respectively, while team C had three boys and four girls. Teams A and B of group “2” were identical to group “1,” but team C consisted of five boys and two girls. This study was reviewed and approved by the Human Research Ethics Committee of the University of La Laguna (Spain) in accordance with the recommendations of the Ethics Committee for Research and Animal Welfare, with written and informed consent from all parents of all participants (Declaration of Helsinki).

### Procedure

The game used in the study is a persecution game known as “The Maze.” It was put into practice by two groups formed of male and female students from conventional physical education classes. The ecological grouping was used so as not to interfere in the development of the teaching team’s program. It was the students’ own teachers who were in charge of guiding the implementation of the games. The formation of the teams (in each group) was random, but gender was taken into account to achieve balanced teams. Before starting the game, we made sure that no student had previously played “The Maze.” In a session before the final registration, the same game was put into practice with the groups in order to resolve doubts related to its rules.

The motor game was engaged in by three teams (different colors were used for each team) in a 20 × 20 space, with simultaneous participation of all the protagonists. The objective of the game is to capture all of the players of the two rival sides following the rivalry relationship (A↔B↔C↔A). To do this, when a player is touched on the back, he must sit down on the spot where he was when touched. At that moment, he becomes a prisoner waiting to be released by a player. The player who usually performs the release action is the partner. However, the “non-restriction of the rule” makes it possible that on certain occasions (usually in terminal situations or close to turning all the players of a team into prisoners), they prefer to prolong the game through paradoxical behaviors, such as making pacts or alliances between opposing teams (Navarro, 1995). Thus, the team that manages to keep at least one free player would win the game, provided that the rest of the teams are sitting (prisoners). It must be remembered that it is a game with an ambivalent and stable communication network (Parlebas, 1988) in which the dynamism is constant since it must adjust to a continuous change of friendly and/or enemy alliances.

The player actions were recorded through the use of two cameras so that it would be possible to, in case of doubt in the observers, resort to a second angle of vision. A single recording was made from the beginning to the end of the game. The time of the analysis, agreed with the observers, did not exceed 3 min for each player.

### Data Quality

In order to determine the data quality (Morillo et al., 2017), inter-observer and intra-observer reliability and validity tests were carried out. Once the observers had uploaded the video to the Lince program, they started to record, separately. When identifying a category, they pressed the button to record. Generalizability analysis was used to estimate accuracy, validity, and reliability (Cronbach et al., 1972; Blanco-Villaseñor et al., 2014). Also, Pearson and Spearman’s correlation coefficients were used.

The values reached always exceeded values of 0.95, thus indicating a high correlation between the different measurements performed by two observers (inter-observer), and two different times (intra-observer). On the other hand, when applying the theory of generalizability, a variance no greater than 1% was evidenced by adding inter- or intraobserver percentages. In this way, the quality of the records was verified.

### Variables, Data Analysis, and Materials

#### Study Variables

1. Roles and subroles: subroles are the minimum units of relational expression, grouped and operationalized from four roles (persecutor, dodger, prisoner, liberator). Each of the four roles was shelled by their respective subroles (Table 1). This mixed registry system was used previously (Pic et al., 2018).

**TABLE 1:** Registration system (4 criteria and 15 categories nested within the criteria).

**Criterion**	**Category**	**Description**
Catcher (C)	CA	Catches an opponent
Catcher (C)	PA	Chases an opponent
Catcher (C)	DEF	Defends a prisoner
Catcher (C)	P	Passivity
Catcher (C)	ALZAAC	Alliance with adversary
Dodger (E)	EA	Dodges an opponent
Dodger (E)	HA	Runs away from an opponent
Dodger (E)	AC	Helps a fellow escape
Dodger (E)	DLL	Moves to free places
Dodger (E)	NR	Does not recognize being caught
Dodger (E)	ALZAE	Alliance between dodging adversaries
Prisoner (P)	A	In attention
Prisoner (P)	CE	Changes position to make release easier
Liberator (L)	TUFC	Touches a prisoner (fellow prisoners)
Liberator (L)	TUFA	Touches a prisoner (adversary prisoners)

2. Gender: girls “G,” and boys “B.”
3. Group: represented by the two groups studied, “1” (composed of three teams) and “2” (composed of three teams). Thus, the groups were considered different statistical units that competed separately.

#### Data Analysis

For all the analyses in this study, we started from statistical levels acceptable in social sciences (*p* < 0.05), except when using THEME (*p* < 0.005). Three phases of data analysis were applied, grouped into (i) descriptive-associative analysis (Chi-square), (ii) supervised learning models (decision trees: CTR), and (iii) T-patterns (THEME).

In the first phase, crosstabulation (chi-square) was applied to verify the existence of an association between the gender variable and the roles. Subsequently, Cramer’s V statistic was applied to calculate the effect size. The previous analysis was then repeated but between the gender and the study groups. In the second phase, decision trees were applied, specifically the CTR model, to reveal the interactive process of the data using the variables of the game, such as the roles (dependent variable), while the predictive variables of the model were gender and group. The predictive tree is a regression-classification model that started from parent nodes with a minimum composition (*n* = 100) and child-nodes (*n* = 50). Finally, in the third phase, the THEME analysis technique was applied to reveal temporary regularities between girls and boys when practicing TMG. THEME is a data-reduction technique. According to Magnusson (2000, pp. 94–95) “if A is an earlier and B a later component of the same recurring T-pattern, then, after an occurrence of A at *t*, there is an interval [*t* + *d*1, *t* + *d*2] (*d*2 ≥ *d*1 ≥ *d*0) that tends to contain at least one occurrence of B more often than would be expected by chance.” The search parameters of T-patterns included for the analyses were significance levels 0.005 and a minimum of two motor occurrences. The search was also addressed in the observational registers of the behaviors (TUFC) and (TUFA) to determine their involvement in the identification of T-patterns.

#### Materials

Lince software (Gabín et al., 2012) was used to manually record the motor actions. The tools used to assess the data quality were the Generalizability Study GT program, v.2.0E (Ysewijn, 1996) and the SAS 9.1 (SAS Institute Inc, 1999) statistical package. The analyses were completed with IBM SPSS 24 (IBM Corp, 2016) and THEME v6 (Magnusson, 1996, 2000; Casarrubea et al., 2015).

## Results

The descriptive and associative results from crosstabulations showed gender differences focused on some specific game roles (*p* = 0.033). Specifically, male dodgers in both groups exceeded (*n* = 107, residual adjusted, 2.5) the female dodgers (*n* = 58, residual adjusted, −2.5), while it was the other way around when examining the captive role. The girls slightly exceeded (*n* = 87, ar, 2.2) boys under the prisoner role (*n* = 85, ar, −2.2). On the other hand, we must also bear in mind that the decision total (*n* = 679) favored boys (*n* = 384) over girls (*n* = 295). Although this total value was not statistically significant, the boys developed more subroles related to the capturing role and the liberating role, being the first in capturing boys (*n* = 119) and capturing girls (*n* = 87) while the releases made by boys (*n* = 73) again exceeded girls (*n* = 63). Finally, the effect sizes found should be considered small (0.114) (Cohen, 1988).

When contrasting the relationship between gender and group, there was no significant association (*p* > 0.05). In spite of this, and the positive or negative values of adjusted residuals not being surpassed (1.9), group “1” reached fewer decisions in its practice (*n* = 294) than group “2” (*n* = 385). In the “1” group, the girls (*n* = 140) made fewer decisions than the boys (*n* = 154), also evidenced in the “2” group in girls (*n* = 155) and boys (*n* = 230).

**FIGURE 1:**
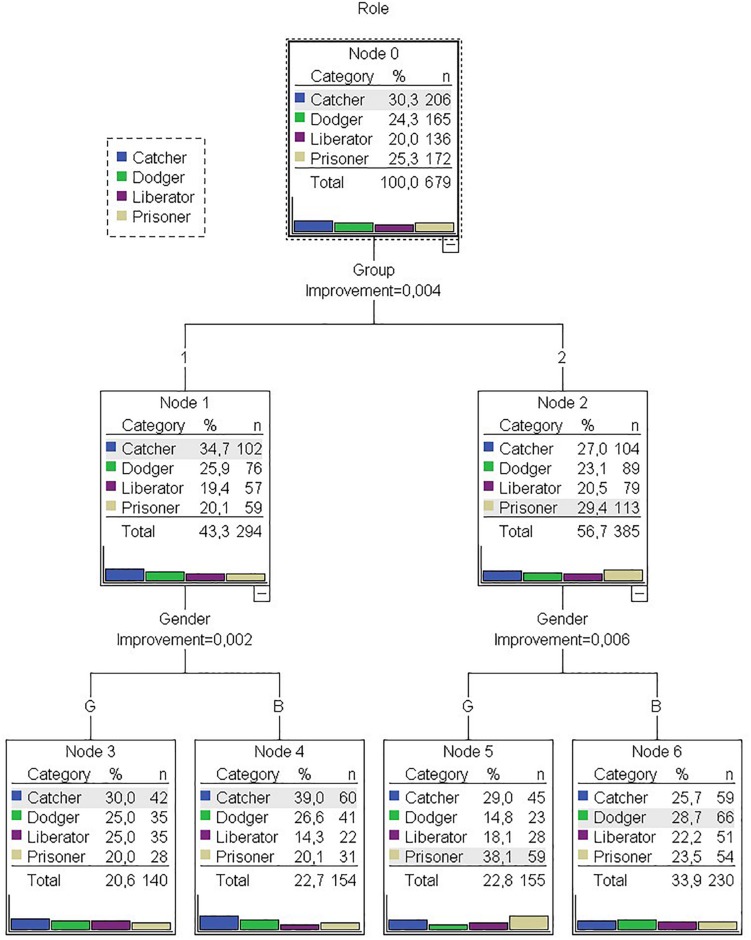
Tree of predictive variables (IV): gender (girl “G” boy “B”), group (“1” “2”), taking into account the role (DV): catcher/dodge/liberator/prisoner.

Figure 1 shows the output of the regression and classification model of the predictive variables gender (girl/boy) and two independent groups (“1,” “2”) formed by three teams each as a function of the variable roles. The role most used by both groups was the catcher (*n* = 206, 30.3%), in second position was prisoner (*n* = 172, 25.3%), then dodger (*n* = 165, 24.3%), and finally, liberator (*n* = 136, 20%). The first partition was established to form node 1 (*n* = 294, 43.3%), with the catcher role being the most used (*n* = 102, 34.7%), while the most used role in group “2” (*n* = 385, 56.7%) was prisoner (*n* = 113, 29.4%). Thus, it was possible to show that, in group “1,” there were fewer behaviors than in group “2”.

On the other hand, the formation of nodes 3–4 in group “2” was distinguished according to gender, as happened with nodes 5–6 in group “2.” Node 3 (*n* = 140, 20.6%), corresponding to girls (G), contained fewer behaviors than node 4, belonging to boys (B) (*n* = 154, 22.7%). Although the most used roles in both boys and girls corresponded to the catcher role, the least present in girls was the prisoner role (*n* = 28, 20%), while, in boys, it was the liberator role (*n* = 22, 14.3%). Regarding now group “2,” girls (G) in node 5 (*n* = 155, 22.8%) achieved less behavior with boys (B) (*n* = 230, 33.9%). Contrary to what happened in group “1,” the role most used by girls (G) was the prisoner (*n* = 59, 38.1%), while, in boys (B), it was the dodger (*n* = 66, 28.7%).

**FIGURE 2:**
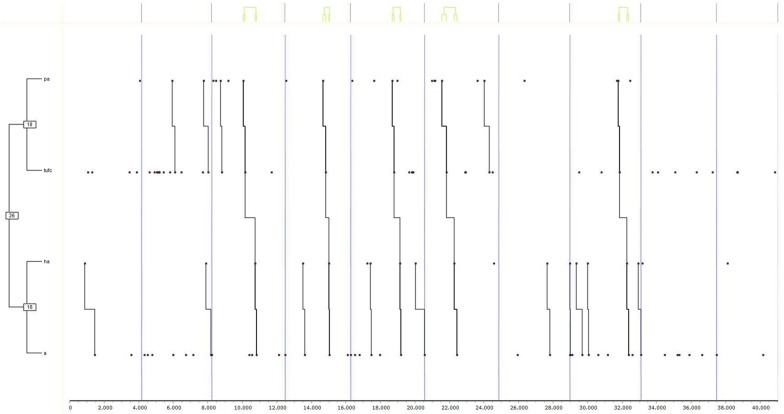
Dendogram obtained from THEME belonging to group “1” exclusively in girls. The decisional categories of the mixed registration system (Table 1) were used for searching for T-patterns.

**FIGURE 3:**
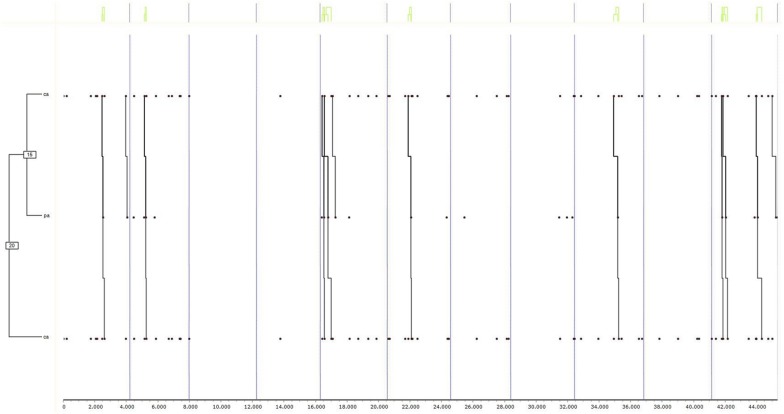
Dendogram obtained from THEME belonging to group “1” exclusively in boys. The decisional categories of the mixed registration system (Table 1) were used for searching for T-patterns.

**FIGURE 4:**
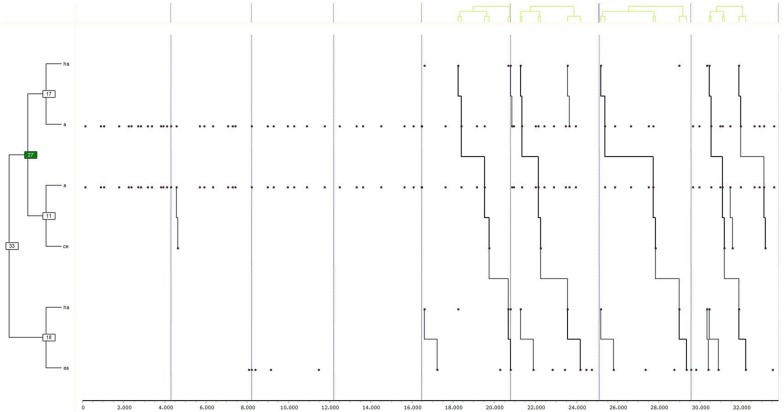
Dendogram obtained from THEME belonging to group “2” exclusively in girls. The decisional categories of the mixed registration system (Table 1) were used for searching for T-patterns.

**FIGURE 5:**
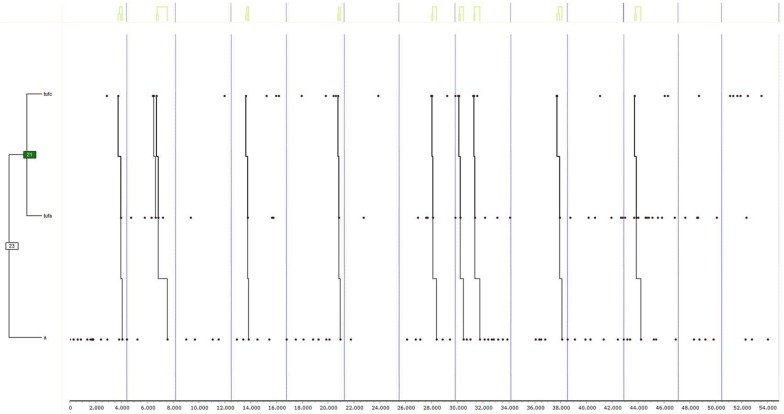
Dendogram obtained from THEME belonging to group “2” exclusively in boys. The decisional categories of the mixed registration system (Table 1) were used for searching for T-patterns.

Finally, the results of the tests are shown first for group “1” in girls (Figure 2) (a) and boys (Figure 3) (b), and secondly, for group “2,” also in girls (Figure 4) (c) and boys (Figure 5) (d). Due to the interest aroused by the behaviors TUFC and TUFA in the development of triadic games, specific searches were conducted.

**(a) Group 1 Female**

In Figure 2, corresponding to group “1,” the relationship between the pursuit (PA) and the release of fellow players (TUFC) is verified. On the other hand, there was also a link between run away actions (HA) and the prisoner role in attention (A). In turn, these two relationships were linked, because compliance with PA-TUFC often led to the HA-A relationship. A specific search was also carried out to determine the implication of the categories TUFC and TUFA in 50% or more of the T-patterns found in this and other dendograms. In this case, the decision to release fellow players (TUFC) was found in group “1” and between the girls, with percentages in the T-patterns of at least 50%.

**(b) Group 1 Male**

On the other hand, the boys in the same group, “1” (Figure 3), alternated between the actions of capture (CA) and pursuit (PA) and both, with more capture actions (CA), which emphasizes the finding that while the boys mostly used the catcher role, the girls showed a greater variability in role, that is, they developed more intensely the four roles described in the mixed registration system. On the other hand, the same specific search that was carried out previously in girls in relation to the implication of decisions TUFC and TUFA in the formation of T-patterns with percentages of 50% or more showed the non-existence of T-patterns.

**(c) Group 2 Female**

In Figure 4, corresponding with group “2,” it was evident that the girls used all the roles except for the catcher role. Thus, the actions of flight (HA) were related to being taken prisoner (A), while the second group of actions carried out by girls related falling prisoner (A) to making spatial movements to facilitate release (CE). Finally, the flight (HA) was also related to the actions of dodging (EA) to get out of difficult situations. Mostly, the relationship was very intense between the first group of behaviors (HA-A) and the second (A-CE), all being related to the last binomial (HA-EA). Therefore, it was found that the girls developed the roles of dodgers and prisoners. Thus, in group “2,” the girls expanded the repertoire of roles used with respect to the “1” group. Minimal percentages of 50% of the records were found for the formation of T-patterns through decisions TUFC and TUFA.

**(d) Group 2 Male**

Liberation behaviors emerged in boys in the second group (group “2”) through the actions of liberation of peers (TUFC) and then release of opponents (TUFA). Finally, after performing the above actions, the players became prisoners in attention (A). Therefore, the boys in group “2” expanded the number of roles used, as was found in the girls in the same group. The specific search for decisions TUFC and TUFA was repeated, confirming the participation of both in a minimum of 50% of the records for the formation of the T-patterns.

## Discussion

The study addressed, through mixed methods, the motor responses of girls and boys when playing TMG. This methodology served to make feasible a study in full contextualization and playful spontaneity ruled by the integration of approaches (Bazeley, 2009). In this way, an approach to a complex triadic reality is proposed. The rule is the main reason for the use of roles (criteria)-subroles (categories) through which to achieve intentional strategic development and interest in winning the game. The identification of the system of categories, derived theoretically from the game roles, has been guided by the internal role-logic relationship of the game (Parlebas, 1981, 1988), allowing a reduction in complexity (Balagué et al., 2013).

It is this internal order when developing the game, in addition to the relational enrichment provided by the triad, that has enabled us to search for the behavior of the descriptive variables and the outcrop of T-patterns through which to judge gender differences. Attached to the context, but oblivious to the research carried out, it is evident that the playful preferences of the ages throughout childhood, in this type of game, already mark the gender behavior at later ages as analyzed; in addition, a current play culture that channels competition through sport with its consequences also related to gender is added to this context.

Among the most remarkable results, there was unified statistical backing linking triadic play with the construction of gender. However, it was through the use of THEME that the analyses with the highest statistical consistency (*p* < 0.005) and methodology were obtained, due to the inclusion of the temporal dimension in the TGM decision complex as a whole (Araújo et al., 2014). The emergence of temporary chains of subroles dissipates doubts, showing a high specialization of the boys on the roles (and their corresponding subroles) leading to direct victory in the game, while the girls showed a more balanced distribution in role selection in the game. What apparently would be a larger form of participation in girls contains subsidiary decisions if possible adaptive subroles are nested under the second observational macrocriterion or role (Parlebas, 1981) of “catcher.” That is, the subroles played by the girls are more widely distributed than those of the boys.

We found, in the present study, small size effects in the use of subroles according to gender. Similar studies found superior effects when the decisional effectiveness of boys on girls (effect size *d* = 0.27) (Pic et al., 2019) in shared-space and ball-throwing motor games is pointed out. At this point, one could ask about the play preference for playing certain games because of gender. In this sense, it was confirmed that while girls and boys preferred persecution games, fighting games belonged only to the male preferential spectrum (Smith et al., 1992). Following Pellegrini (1995), in persecution games, there is no transmission of a hegemonic power, which does exist in fighting sports, thus validating the use of pursuit games as a means to identify motor asymmetries, since they do not perpetuate the established order of male supremacy. While it is true that these forces prevalent in both genders are less pronounced in the game of persecution, it is not exempt from them. The reference culture has demonstrated its influence on different human activities (Lévi-Strauss, 1987); therefore, it would be appropriate to point out that culture could be behind the responses found in girls and boys (Costa et al., 2001; Wood and Eagly, 2002) through the predominance of gender stereotypes.

Assuming differences in players at the end of childhood by aged, it is not negligible that the boys and girls studied already start with differences in the ludic preferences that they have lived. Sluckin (1981) warned that the commitment of boys in capture games was four times the commitment of girls (Sluckin, 1981). This author attributed greater competitive emphasis to boys with respect to girls, which could be explained by means of a certain vigorousness necessary to participate in this type of game (Fabes, 1994). In this same sense, the differences found by Navarro (1995, 2009) in 10-year-old girls, in relation to boys, in the original version of the persecution game “The Maze” would invite us to think that the playful preferences were already appreciable at early ages and had an extension at later ages. In other words, there may be a desire to compete in boys and to feel more perseverant in the reduced decisional practice and specialization of roles in a preferred game in the gender differences that have been obtained. All this contributes to telling us that TMG is a game structure where relational differences are expressed, as are gender ones, and that the triad causes them to emerge due to having a greater spectrum for these relationships.

Perhaps the sign of valence (Heider, 1946) of the communication relationship could help in understanding the distribution of the specific use of roles (Pic and Lavega-Burgués, 2019) in the game “The Maze,” because, under the effect of the paradoxical situation and in actual practice, the role has a positive valence when applied cooperatively and a negative valence when acting antagonistically. It is not only a matter of the role-role relationship when playing; it is a product of the situationality (Gómez et al., 2013) and the interactive solution found by the players (adversaries or partners). The study presented has been able to satisfactorily detect this by means of descending to the minimum level of behavioral unit of the subrole (Parlebas, 1981, 1988) while at the same time identifying the antagonistic or cooperative use of the paradoxical role.

While the girls used almost all the roles in both groups, which could be described as more complex, the boys reduced their practice to perform capture and salvation actions in the second group. Perhaps the behavior of boys responded to exclusively strategic reasons, as a means to achieve victory, while the girls showed themselves more inclined to consider other influences of a playful and systemic nature and, therefore, be more harmonious in the use of other roles. Some clues could be forming a puzzle to weave a complex web of gender behavior. In the classic Vinacke’s (1969) studies of triad coalitions, it was found that girls applied less risky strategies and made unnecessary coalitions to achieve success. This same imbalance, favoring competition on cooperation of the boys was confirmed (Kivikangas et al., 2014) by the girls showing themselves to be more balanced in the cooperation-competition binomial.

In the same study, the boy’s ability to cooperate was also recognized; this is analogous in our study to the use of the liberator role identified by THEME in group “2” but not in group “1,” given that only release actions greater than 50% (TUFC) were identified in girls. Although the cooperation did not rest exclusively on girls, they were more attracted to it (cooperation) than were boys, confirming positive emotions after their experimentation in a context of motor games (Lavega et al., 2014b) extrapolated to other contexts (Johnson and Engelhard, 1992; McKay et al., 2000). Positive emotions in favor of cooperation in girls (Lavega et al., 2014b) find their preferential parallelism in rivalry (Lavega et al., 2014a; Muñoz et al., 2017).

The boys, when selecting their game solutions, have followed the most important strategic motive, to win, which is the most advantageous solution as a game achievement. Keeping the distances, this same strategic advantageous solution is behind the attraction for the rivalry to which Kivikangas et al. (2014) alluded in boys. Also, in a context of motor play, greater proportions of male protagonism and ball throws (Gutiérrez and García-López, 2012) were identified. Thus, it can be understood that the logical development of the boys in the game “The Maze” was to use the “catcher” role to win and only in case of necessity to use the “dodger” role as strategic premises. The girls, on the other hand, developed a variety of roles and subroles linked to tasks secondary to the main strategic achievement of the game; in this sense, the true weight of contextual variability in TMG was more assimilated by girls, although with this strategic disadvantage. Thus, the adaptations to the new triadic environment (Pic et al., 2018) directly refer more to the girls, as it also refers to them the properties and specificity nested in the dimension of transience (temporal) and its correlation in the use and importance of time in THEME. That is, the girls show a greater adaptive repertoire than the boys in their strategic itineraries, while the boys remain more in the role of strategic dominance of the game.

The events derived from the development of the game have not been an obstacle to the analysis model, finding gender differences in separate groups, since the “role” criterion is a sufficiently relevant element. Thus, the regression and classification model (CTR) reflected the group variable in the first place to segment the records, when both groups were divided according to gender. All this depended on the dependent variable role. Although these results show that there were decisional differences when playing between girls and boys, it also indicates that the “1” and “2” groups had a collective identity. It seems that the group behavior and the adaptation of the groups to the triadic situations describe an autonomy of the groups in interaction, but at the same time, there is a different adaptive evolution between the groups. That is why the behavioral variability of the individual adapts socioculturally to the community, which makes it difficult to perform suitable calculations; however, gender differences emerge when they are analyzed with adequate methodological approaches.

The relevance of this study can be summarized in two questions: (a) the evidence of gender-related strategies through THEME that are hidden from the human eye and about the practice of TMG is relevant; (b) the identification of girls’ decisional variability is comparatively extended in terms of role performance with respect to boys. Operationalizing a motor game played in confrontation under the criterion of the role links a theoretical construct with a methodological criterion, because to enhance the quality analysis is to establish the maximum closeness between the theoretical interpretation of the game and the methodological coherence.

Among the limitations of the study, it is worth mentioning the increase in the number of participants as one of the greatest challenges or the increase of time to record playful events (observable behaviors). It would also be interesting to replicate this work in other cultures (Wood and Eagly, 2002) contexts, to see if the differences reproduce again. It would be complex to ensure that the physical development of girls and boys has been neutralized (identical) when they were playing. Though logical, it is not appropriate to exclude participants from a physical education class during official hours, as it constitutes a legal impossibility. On the other hand, finding perfectly gender-balanced groups could be indicated. In addition, it would be convenient to develop different recording tools for the study of motor games, more specifically TGM, because they contain a development of an internal logic of situations. The study of TGM is in an embryonic phase, but different pedagogical applications could be detached from its use, trying to address more transitory decisional states among other differential elements with respect to the best-known play culture. Interdisciplinary study of TGM from a gender perspective might be appropriate; that is, in terms of both methodological and disciplinary approach. Awakening the interest of other research groups for TGM would complete the interpretive circle of the triad and how gender differences affect the decision-making complexity depending on spatial criteria among others not addressed by this work.

## Conclusion

The viability of the study of gender differences when playing TMG through mixed methods was verified. There was greater masculine participation in the catcher, dodger, and liberator roles, while the girls used the prisoner role more than boys in TMG (Figure 1). Groups “1” and “2” showed a certain group singularity reflected in the decision tree and in the analysis of T-patterns. In group “2,” the TUFC and TUFA behaviors of girls and boys exceeded 50% of the records in the form of T-patterns. However, TUFC was exclusive in girls in the group “1.”

The most relevant conclusion was based on the temporal dimension. Greater complexity (quantity and variety according to THEME analysis) of roles-subroles was identified in girls (1CA, 3HA, 1EA, 3A, 1CE, and 1TUFC) in the situational dynamics of TMG, while it was lower in boys (2CA, 1PA, 1A, 1TUFC, and 1TUFA). The most gender-specific differences revealed that boys were more seduced by the catcher (CA, PA) and liberator (TUFA) roles; while girls developed a decisional approach in the roles dodger (HA) and prisoner (A, EA) subject to the TMG play system.

## Data Availability Statement

The datasets generated for this study are available on request to the corresponding author.

## Ethics Statement

The studies involving human participants were reviewed and approved by Ethics Committee for Research and Animal Welfare. Written informed consent to participate in this study was provided by the participants’ legal guardian/next of kin.

## Author Contributions

MP and VN-A have contributed to the theoretical and methodological development of the manuscript while GJ and MP have contributed with the data analysis. MP, VN-A, and GJ have prepared results and discussion.

## Conflict of Interest

The authors declare that the research was conducted in the absence of any commercial or financial relationships that could be construed as a potential conflict of interest.
